# Forced Degradation Behaviour of Fluphenazine Hydrochloride by LC and Characterization of its Oxidative Degradation Product by LC–MS/MS

**DOI:** 10.3797/scipharm.1411-04

**Published:** 2014-12-22

**Authors:** Kashyap N. Thummar, Dilip J. Ghava, Anvi Mistry, Ashish Vachhani, Navin R. Sheth

**Affiliations:** Department of Pharmaceutical Sciences, Saurashtra University, Rajkot – 360005, Gujarat, India

**Keywords:** Fluphenazine hydrochloride, Force degradation study, HPLC, Stability indicating assay, LC-MS/MS

## Abstract

A novel, stability-indicating high-performance liquid chromatographic (HPLC) method is delivered for the determination of fluphenazine hydrochloride (FPZ) and its degradation products. The forced degradation testing of FPZ was carried out for hydrolytic, oxidative, photolytic, and thermal degradation. The degradation appeared using a reversed-phase C18 column at ambient temperature with a mobile phase comprised of methanol : acetonitrile : (10 mM) ammonium acetate (70:15:15, v/v/v) pH 6.0, adjusted with acetic acid, having a flow rate of 1 ml min^−1^ and a detection wavelength at 259 nm. Primarily, the maximum degradation products were formed under oxidative stress conditions. The product was distinguished through LC-MS/MS fragmentation studies. Based on the results, a more complete degradation pathway for the drug could be proposed. The modernized method was found to be precise, accurate, specific, and selective. The method was found to be suitable for the quality control of fluphenazine hydrochloride in the tablet as well as in stability-indicating studies.

## Introduction

**Fig. 1:**
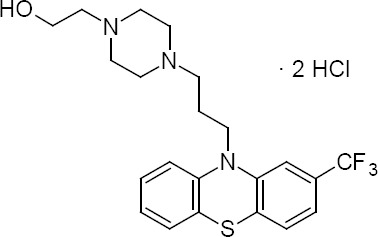
Structure of fluphenazine hydrochloride

Fluphenazine hydrochloride (FPZ) is an antipsychotic drug which blocks postsynaptic mesolimbic dopaminergic D1 and D2 receptors in the brain. It is used for the treatment of schizophrenia and bipolar disorder [1]. Fluphenazine is a trifluoromethyl phenothiazine derivative with a piperazine side chain and its hydrochloride salt is used for oral administration. Fluphenazine hydrochloride is chemically 2-(4-{3-[2-(trifluoromethyl)-10H-phenothiazin-10-yl]propyl}piperazin-1-yl)ethan-1-ol dihydrochloride (Fig. 1). It is a white crystalline, odourless powder which melts in the range of 226–233 °C [2]. Fluphenazine is official in IP [3] and BP [4].

The chemical and physical stability of FPZ has been studied by several workers. It is stated to be fairly stable when stored in closed containers, but shows instability in open containers and upon exposure to high temperature and stress conditions. Numerous analytical techniques have been noticed in the literature for the analysis of FPZ. The techniques include spectrophotometry [5], spectrofluorimetry [6], turbidmetric [7], gas chromatography (GC) [8], HPTLC [9, 10], etc. A number of high-performance liquid chromatography (HPLC) methods have also been reported for this drug using ultraviolet (UV) as well as mass (MS) detectors [11–16]. A comprehensive LC and LC–MS/MS study of the degradation behaviour of FPZ under various ICH-prescribed stress conditions has been lacking.

The initial literature search indicated that most of the reported HPLC methods for FPZ, indicating official compendia, were developed either on C8, C18, or polymeric columns, despite being the same, the peak shapes were not good and there was considerable tailing. So attempts were made to develop a simple method according to ICH guidelines on the C18 column, with the possible lowering of column temperature. Therefore, it was influenced to carry out forced decomposition studies according to the ICH requirements and develop a selective and validated stability-indicating HPLC method. An integral aim of the study was to identify new degradation products, if any, and to postulate the degradation pathway of the drug by LC-MS/MS.

## Experimental

### Chemicals

Pure fluphenazine hydrochloride was obtained as a gift sample from Centaure Pharmaceutical Pvt. Ltd. (Mumbai, India) and it was used without further purification. Analytical reagent (AR) grade sodium hydroxide (NaOH), hydrochloric acid (HCl), hydrogen peroxide (H_2_O_2_), and HPLC grade acetonitrile (ACN) and methanol were purchased from Merck Chemicals Pvt. Ltd. (Mumbai, India). Buffer salts and all other chemicals were of AR grade obtained from Rankem Chemicals (Faridabad, India). HPLC grade water was freshly prepared using the Milli-Q Synergy UV Apparatus (Millipore, Mumbai) and it was used throughout the study.

### Instrumentation

Photostability was done in a UV-chamber; the photostability UV-chamber was equipped with an illumination bank on the inside top consisting of a combination of one UV lamp and one white fluorescent lamp, all in accordance with ICH guideline Q1B [17]. Both fluorescent and UV lamps were put on simultaneously.

The HPLC system consisted of the Rheodyne manual injector (20 µl), solvent delivery pump (LC2010AD Shimadzu), UV/Visible spectrophotometer detector (SPD-2010A, Shimadzu), and LC Solution software (all from Shimadzu, Kyoto, Japan). A column hibarR C18 (250 mm × 4.6 mm, particle size 5 µm) was used for the LC studies and to develop the stability-indicating assay method (SIAM). The temperature and humidity studies were carried out in a stability chamber (Remi SC-16 Plus, Remi Elektrotenik Ltd.).

The LC–MS/MS studies were carried out on a system in which the LC part consisted of HPLC (Prominence), comprised of an on-line degasser (DGU-20A5R), autosampler (SIL-20ACHT), column oven (CTO-20AC), and PDA detector (SPD-M20A-PDA detector. The whole system was operated using Lab Solution software LC-MS version 5.4 for LCMS-8030 (Shimadzu, Japan). The mass spectrometer was run in positive electron spray ionization (ESI) mode with a mass/charge (m/z) ratio in the range of 50–1000 m/z. A pH/ion analyzer (Eutech Pvt. Ltd.) was used to adjust and check the pH of the buffers and other solutions. Other pieces of equipment used were a sonicator (Jain Scientific), analytical balance (Shimadzu, Japan), and auto pipettes (Accu Biotech, Beijing, China).

### Preparation of Standard Stock Solution

Ten mg of FPZ was weighed and transferred into a 10-ml volumetric flask and made up to volume with HPLC grade methanol to obtain 1000 µg ml^−1^. One ml of the above solution was withdrawn and transferred into a 10-ml volumetric flask and made up to volume with methanol to achieve a 100 µg ml^−1^ concentration and this solution was used throughout the study.

### Degradation Studies

All degradation studies were carried out under the conditions of hydrolysis, oxidation, photolysis, and dry heat as mentioned in the ICH Q1A R(2) guideline [18].

#### Acidic Degradation

For the acid decomposition studies, 1 ml of stock solution and 1 ml of 0.1 N HCl in a 10-ml volumetric flask were taken, respectively, and the volume was made up to 10 mL with methanol and the solution was left for 12, 24, and 48 hours. From that, 1 mL of stock solution was withdrawn and neutralized with NaOH up to neutral pH and then diluted up to 10 mL with methanol. The final solution was analyzed for the degradation study.

#### Alkaline Degradation

For the studies in alkaline conditions, 1 mL of stock solution and 1 ml of 0.1 N NaOH in a 10-mL volumetric flask was taken and the volume was made up to 10 mL with methanol and the solution was left for 12, 24, and 48 hours. From that, 1 mL of stock solution was withdrawn and neutralized with HCl up to neutral pH and then diluted up to 10 mL with methanol. The final solution was analysed for the degradation study.

#### Oxidative Degradation

For oxidative conditions, decomposition was done in 10% H_2_O_2_. We took 1 mL of stock solution and 1 mL of 10% H_2_O_2_ was added and the solution was made with methanol up to 10 mL and left for 12, 24, and 48 hours. Then, we further diluted it with 10 mL methanol, and the final solution was used for analysis.

#### Photolytic Degradation

Photolytic studies were done by using a UV chamber. The drug was kept in a petri dish with as thin of a layer as possible for 3, 5, and 7 days. Then it was analysed.

#### Thermal Degradation

Thermal decomposition studies were performed by exposing a solid sample of the drug to dry heat at 75°C, then kept for 12, 24, and 48 hours and the solution having a concentration of 100 μg mL^−1^ was prepared and analysed at 24, 48, and 72 hours.

### Preparation of Samples for HPLC Analyses

HPLC studies were performed on stressed samples individually (after appropriate dilution). Studies on individual reaction solutions were carried out using methanol-ACN-ammonium acetate (10 mM) (70:15:15, v/v/v) (pH 6.0) adjusted with acetic acid as the mobile phase. The samples (10,000 µg mL^−1^) were diluted 100 times with methanol in the case of acidic (0.1 N), alkaline (0.1 N NaOH), and oxidative (10% H_2_O_2_) solutions. The solid samples were suitably diluted in methanol.

In all HPLC runs, the mobile phase was filtered before analysis through a 0.45 µm nylon membrane and sonicated for 15 min before use. The injection volume was 20 µl and the flow rate was 1 ml min^−1^ for the initial studies. The detection wavelength was 259 nm.

### Separation Studies and Development of the Stability-Indicating Method

First, HPLC studies were performed on all reaction solutions individually, and then on a mixture of degraded drug solutions. A C18 column was employed. Different logical modifications like a change in pH, different mobile phase compositions, and column temperature adjustment were tried to get good separation between the drug and the degradation products, as well as between the degradation products. Water was avoided during the study because of its significant ionization effect on the drug.

### Validation of the Developed Method

Validation was done with respect to various parameters, as required under ICH guideline Q2(R1) [19]. To establish linearity and range, a stock solution containing 100 µg ml^−1^ of the drug in methanol was diluted to yield solutions in the concentration range of 10–60 µg ml^−1^. The intraday and interday precision were established by analyzing 40 µg ml^−1^ drug solutions in six replicates on the same day and on consecutive days, respectively. To determine intermediate precision, the brand of the column was changed and also the whole experiment was conducted by a different person. Accuracy was determined by spiking a test drug sample (40 µg ml^−1^) with three known concentrations of the std. drug, viz., 32, 40, and 48 µg ml^−1^ in triplicate and then determining the percent recovery of the added drug. The specificity of the method was established by determining the peak purity using a PDA detector. Also, the resolution factor for the drug and the nearest resolving peak were determined. In fact, both peak purity as well as the resolution were determined for all the degradation products’ peaks, in addition to the drug peak, to prove that the developed method was selective in nature.

### Development of the LC–MS/MS Method and Characterization of Degradation Products

To characterize degradation of a product by LC–MS/MS studies, the developed method was used LC-MS/MS. Apart from the flow rate, the rest of the parameters were the same. Satisfactory separation of degradation products were achieved using a C18 column (250 mm × 4.6 mm and particle size 5 µm). The obtained m/z values in positive ESI mode were used to identify the degradation product. The fragmentation pattern was also investigated. Based on the mol. wt. and the fragmentation pattern, structures could be proposed for the unknowns. The degradation pathway was outlined based on the results.

## Results and Discussion

Validation of the Developed Stability-Indicating Method:

### Linearity

The response for the drug was found to be strictly linear in the investigated concentration range. The values of the area under the curve and concentration are given in Table 1. The *r*^2^ value was equal to 0.9991.

**Tab. 1:**
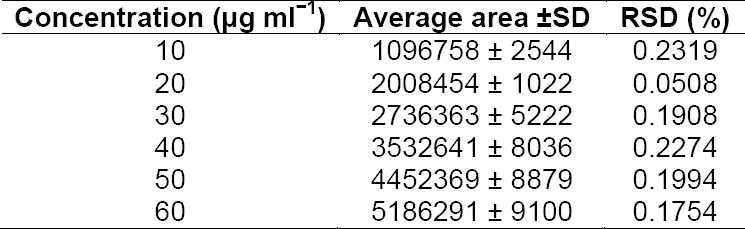
HPLC data of FPZ from linearity studies (n=3)

### Precision

Data obtained from the precision experiments are given in Table 2, the data for intraday and interday precision studies at six replicate concentrations of 40 µg mL^−1^ were in the linearity range. The %RSD values for intraday and interday precision were <2%, indicating that the method was sufficiently precise.

**Tab. 2:**
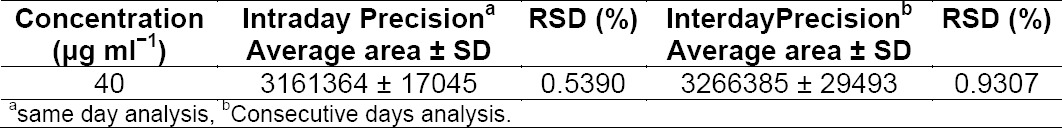
HPLC data of intraday and interday studies (n=6)

### Accuracy

As shown from the data in Table 3, good recoveries of the drug in the range from 99.54% to 100.13% were made at various added concentrations, despite the fact that the drug was fortified to a mixture that contained the drug as well as the test formulation.

**Tab. 3:**
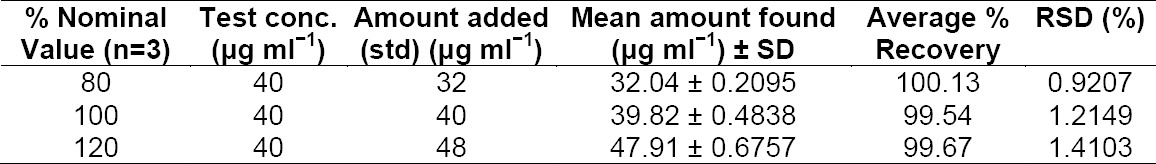
HPLC data of recoveries of FPZ (n=3)

### Specificity

The specificity of the method was established by verifying the purity of the drug peak in a mixture of stressed samples by PDA analyses. The purity angle (PA) value 0.999957 for the drug peak in a mixture of stressed samples was found to be less than the purity threshold (TH) value 1.000000, indicating the absence of any co-eluting peak in the drug peak. The resolution factor for the drug peak was >2 from the nearest resolving peak. The validation parameter is summarized in Table 4.

**Tab. 4:**
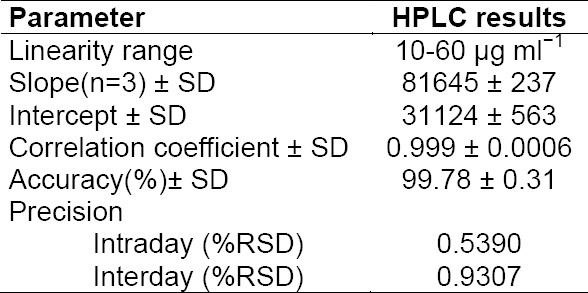
Summary of validation parameters

### HPLC Studies on the Stressed Solutions

There was significant degradation observed in acidic, alkaline, photolytic, and thermal degradation with different strengths of acidic alkaline. But the degradation peak was not observed in PDA detection due to one of its possible reasons that the degrading product might have been non-chromophoric in nature or in a very low quantity. In the case of only oxidative stress degradation conditions, the degradation peak was found significantly and therefore it was only proposed for the confirmation of the molecular structure and formula by LC-MS/MS.

FPZ degraded to one or two degradation products in the oxidative stress conditions, HPLC studies on FPZ under different stress conditions using a mobile phase composition of methanol-ACN-ammonium acetate (10 mM) (70:15:15, v/v/v) (pH* 6.0), adjusted with acetic acid as the solvent system, suggested the following degradation behavior was interpreted based on the area calculation with a comparison of the standard peak area.

### Acidic Conditions

**Fig. 2:**
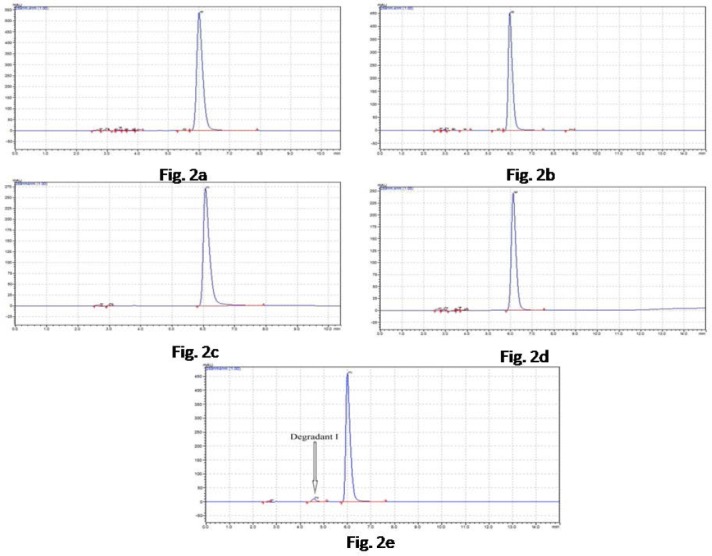
Representative chromatogram of HPLC: 2a) Chromatogram of 0.1 N HCl at 12 H, 2b) Chromatogram of 0.1 N NaOH at 12 H, 2c) Chromatogram of photolytic degradation at 3 days, 2d) Chromatogram of thermal degradation at 75°C at 12 H, 2e) Chromatogram of oxidative degradation of 10% H_2_O_2_ at 12 H

When the FPZ drug was in 0.1 N HCl for 12 H, around 17% degradation was seen, but there was no corresponding rise in degradation product peaks (Fig. 2a).

### Alkaline Conditions

FPZ was found to be labile in alkali, and around 14% degradation of the drug was observed in 0.1 N NaOH at 12 H, but no additional peak was found in the chromatogram (Fig. 2b).

### Photolytic Conditions

Under photodegradation, FPZ was less susceptible to photodegradation. The drug was placed in a UV chamber for 3 days. A level of 6% degradation was found (Fig. 2c).

### Thermal Conditions

Thermal degradation around 12% was seen upon subjecting the drug to dry heat at 75°C for 12 H. There were no degradation peaks found, but the peak area of the drug peak was decreased (Fig. 2d).

### Oxidative Conditions

FPZ was found to be degraded in 3% H_2_O_2_ at room temperature. However, almost 33% of drug degradation was seen upon exposure to 10% H_2_O_2_ for 12 H at room temperature. Only one small peak was seen in the chromatogram (Fig. 2e).

All the stress conditions have been studied and the % drug degradation is shown in Table 5.

**Tab. 5:**
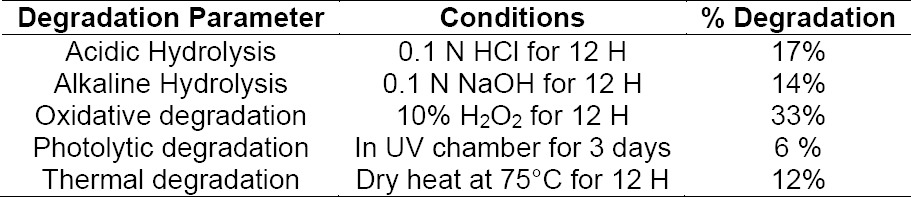
HPLC summary of the degradation studies

### Development of the Validated Stability-Indicating HPLC Assay Method Development and Optimization of the Method

Subsequent trials were made on stressed samples using phosphoric acid in water of various pHs and ACN and methanol at various temperatures. The peaks for the drug and degradation products were not well-separated or did not have an acceptable shape at pH >3. The best separation was achieved on the C18 column (hibarR, 250 mm × 4.6 mm, 5 µm) at room temperature using a mobile phase composed of methanol-ACN-ammonium acetate (10 mM) (70:15:15, v/v/v) (pH* 6.0), adjusted with acetic acid, which was run in isocratic mode. The flow rate was 1 mL min^−1^ and the detection wavelength was 259 nm.

### Stability-Indicating Nature of the Developed Method

The method was able to resolve the oxidative degradation product of the stressed sample. The peaks of the oxidative degradation product were not only well-resolved from the drug, but also from one another. The method thus proved to be selective and stability-indicating.

**LC–MS/MS Studies on Oxidative Decomposition Samples of FPZ**

**Fig. 3:**
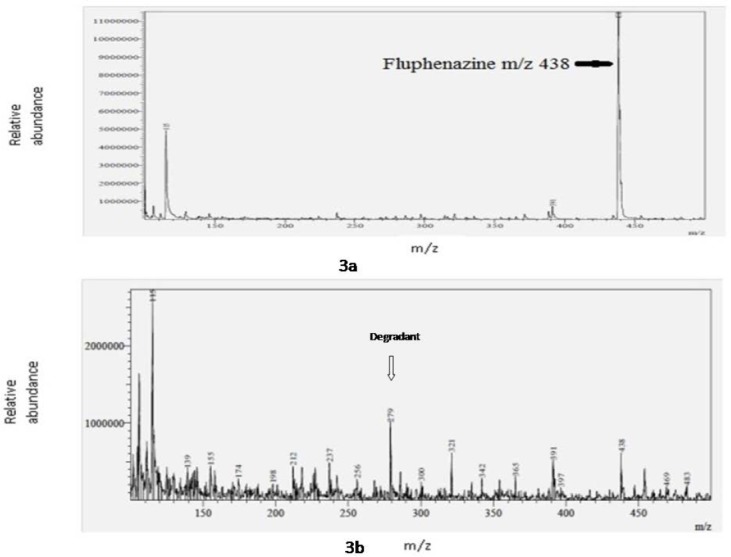
a) Mass spectra of fluphenazine hydrochloride, b) Mass spectra of the unknown degradation product.

There were no changes in the conditions of HPLC and LC-MS/MS except for flow rate and appropriate mass tuning. The mass scan range was selected to be 100–500 dalton. The obtained m/z values in positive ESI mode were used to identify the degradation product. The collision energy applied was 25 eV. Total run time was 5 min. The precursor ion peak of the standard FPZ was observed at m/z 438. The mass spectra of the drug and degradation products are shown in Fig. 3a & 3b. The observed m/z 438 values for the molecular ion peak and major fragments of the drug and degradation products were m/z 391, 321, 279, and 115. From these, the values m/z 438 and m/z 279 are matched with the molecular weight and fragmentation scheme of the degradation product of the drug. According to the m/z values and fragmentation pattern, the structures for the remaining degradation product could be proposed.

**Proposed Degradation Pathway of the Drug**

**Fig. 4:**
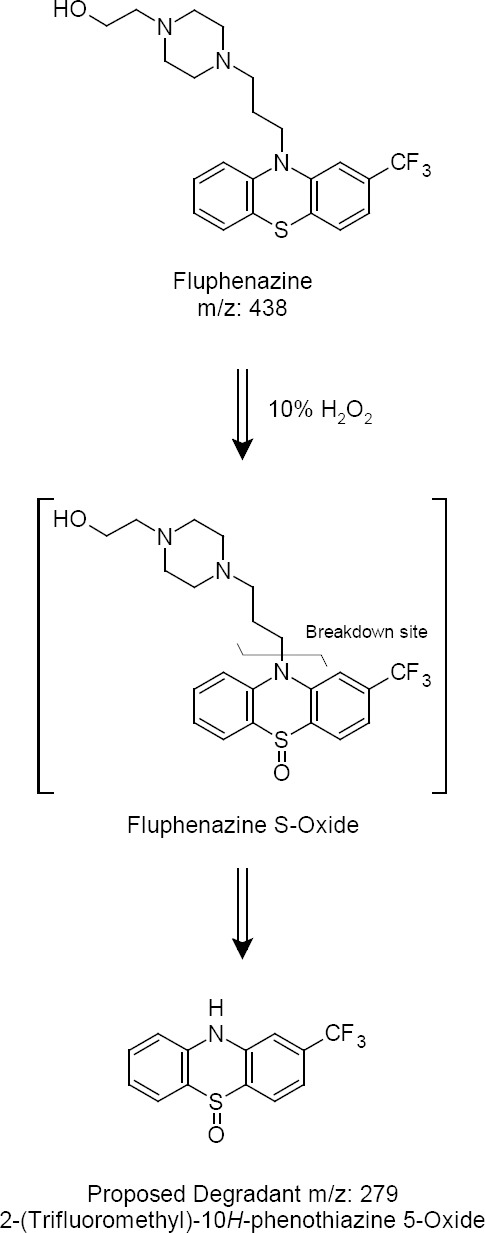
Proposed oxidative degradation pathway

It is known that FPZ undergoes oxidation to give the oxidative product. The route of decomposition to these products is outlined in Fig. 4, which also includes the pathway for the generation of the unknown product.

The decomposition route is explained on the basis that the FPZ drug mass spectra shows m/z 438 and the degrading mass spectra shows m/z 279. So, these m/z ratios were found when there were additions of oxygen to the parent ring and removal of a side chain giving the value m/z 279. FPZ has two vulnerable sites where oxygen gets attached, one is nitrogen and the other is sulphur. Oxygen has more of a chance to attach to sulphur because sulphur has an open site where oxygen can attach and sulphur has the tendency to form oxides in the presence of oxygen. The oxygen has a lone pair of electrons which is accepted by sulphur and it forms a double bond. The whole scheme in Fig. 4 seems to be logical, as clearly the additional products are formed following a parallel reaction pathway to the already known degradation products of the drug.

## Conclusion

In this study, it was possible to develop a selective and validated stability-indicating HPLC assay method for fluphenazine hydrochloride on a C18 column, which could separate the drug and its degradation products formed under oxidative forced degradation conditions. FPZ was found to be unstable in the solution state, whereas it was comparatively much more stable in the solid-state. Forced degradation at different time intervals and in different conditions gives information regarding the degradation kinetic behavior of FPZ.

The m/z values and fragmentation patterns obtained for the degradation products through the LC–MS/MS studies helped to confirm the presence of the known products and to propose the structures of unknown compounds. The results in totality helped to draw out a more extensive degradation route of the drug.

Indirectly, the study highlights the benefit of the use of the ICH forced degradation testing approach in the establishment of complete degradation pathways of drugs. It is hoped that this report on a stability-indicating method and degradation route of FPZ would be helpful for the multiple generic manufacturers of the drug around the globe by saving them from unnecessary repetition of the same studies.
